# Cognitive impairment among older adults in India: understanding the role of substance use and lifestyle factors

**DOI:** 10.1186/s12877-025-06650-w

**Published:** 2025-12-01

**Authors:** Amir Ali, Manas Ranjan Pradhan

**Affiliations:** 1 International Institute for Population Sciences, Govandi Station Road, Mumbai, 400088 India; 2 Fertility and Social Demography, International Institute for Population Sciences, Mumbai, 400088 India

**Keywords:** Cognitive impairment, Older adults, Substance use, India

## Abstract

**Background:**

Cognitive impairment represents an emerging significant public health concern and a prominent risk factor for Alzheimer's disease and dementia among older adults. This study examines the association of substance use and lifestyle factors with cognitive impairment among older adults in India.

**Method:**

Utilizing data from the Longitudinal Aging Study in India (LASI) wave 1 (2017–18). We performed bivariate analyses to show association between predictors and outcome variables. Binary logistic regression was employed to understand the impact of substances use, and life style factors controlling the effect of demographic, socio-economic, and health factors on cognitive impairment.

**Results:**

In India, 10.76% of older adults exhibit cognitive impairment. The individual who consumed alcohol were 17% more likelihood to experience cognitive impairment (OR:1.17,95% CI:1.07,1.28). Similarly, those who smoked tobacco were 7% more likely to experience cognitive impairment (OR:1.06,95% CI:0.76,1.15). Life style also plays a significant role in determining cognitive impairment.

**Conclusions:**

Substance use and other lifestyle factors are significantly associated with cognitive impairment among the older adults in India. Therefore, encouraging older adults who use tobacco or alcohol to assess their cognitive function at an earlier stage that could prevent or mitigate cognitive impairment, thereby enhancing their quality of life. This contribute to achieve of Sustainable Development Goals, particularly Goal 3 that guaranteeing good health and wellbeing for all.

## Introduction

Cognitive impairment is a transitional stage between normal aging and dementia, impacting memory and thinking, and posing a public health concern [9]. Cognitive impairment is an early decline in brain function, often a precursor to dementia, which is a group of conditions, including Alzheimer’s that affect memory, reasoning, and daily functioning. Alzheimer's disease and dementia were ranked fifth top ten global causes of death in 2016, reflecting their significant impact as non-communicable diseases [34]. Globally, cognitive impairment affects approximately 50 million individuals. The prevalence of cognitive impairment is expected to nearly double every 20 years, reaching an estimated 42.3 million individuals in 2020 and 81.1 million by 2040 [45]. The most significant rates of growth are found in India (approximately 33.6%), China, South Asia, and the Western Pacific region. Over 3.7 million people were affected by cognitive impairment in India by 2010, which is expected to double by 2030 [47]. According to the World Population Prospects 2022, the proportion of individuals aged 65 and over is projected to increase from 10% in 2022 to 16% by 2050 [46]. Simultaneously, the aging process and the use of commonly prescribed medications can increase sensitivity to substance use, accelerated the risk of cognitive impairment [13].

There are several societal risk factors that contribute to substance use among older adults [36]. The consumption of tobacco products, such as hookah and beedi, has increased among the older person, due to the social acceptance of these products and their use for purposes of social interaction and relaxation [12]. Although a range of studies has produced controversial result, evidence suggests that light to moderate alcohol consumption may reduce the risk of developing cognitive impairment [18, 27]. Additionally, some study highlighted smoking, and alcohol consumption can exacerbate cognitive disturbances among elderly [3, 21]. Major contributors to this issue include demographic shifts characterized by an increasing older population, as well as adverse socioeconomic conditions such as poverty, limited access to education, inadequate healthcare facilities, and unhealthy lifestyles [8, 32]. Nevertheless, various studies have explored the relationship between substance use disorders and cognitive outcomes [10]. Cognitive impairment significantly associated with general health condition. Cognitive capacity is associated with better mental and physical health outcomes and reduced mortality [29]. Among older person who had never sought treatment, there was significant variability in the progression of symptoms, raising questions about the effectiveness of existing disease progression models [19, 39].

The other risk factors of cognitive impairment include being older female, from minority racial groups, having lower levels of education, income and experiencing social [2, 10, 22]. Additionally, it is necessary to acknowledge the correlation between the rising prevalence of chronic diseases with aging and the decline in certain cognitive impairment in later life [15]. Social factors such as advancing age, lack of education, widowhood, and morbidity are significantly associated with greater cognitive impairment among the older person [38]. Older adults are more likely to abuse legal substances, including alcohol, tobacco, and over-the-counter medications [17]. Alcohol consumption tends to decrease with advancing age. This trend may be attributed to several factors such as the aging effect where physiological changes reduce alcohol intake and the cohort effect which suggests that heavy drinkers may have a higher mortality rate, thereby reducing the number of heavy drinkers in older age groups [43]. Several epidemiological studies have demonstrated that moderate alcohol consumption is associated with a lower risk of dementia compared to non-drinking, paralleling the observed association between moderate alcohol intake and a reduced risk of cardiovascular disease [30]. In the Italian Longitudinal Study on Aging, alcohol consumption, as opposed to non-consumption, was associated with a lower risk of dementia among participants with mild cognitive impairment MCI [41]

India's diverse population and cultural practices profoundly influence the relationship between substance use and cognitive impairment. Despite the increasing prevalence of cognitive impairment in India, there is a notable lack of research examining the specific impacts of substance use and other lifestyle factors on cognitive impairment. This study seeks to address this research gap by investigating the association of substance use and lifestyle factors with cognitive impairment among older adults in India.

## Data and methodology

### Data source

**Fig. 1:**
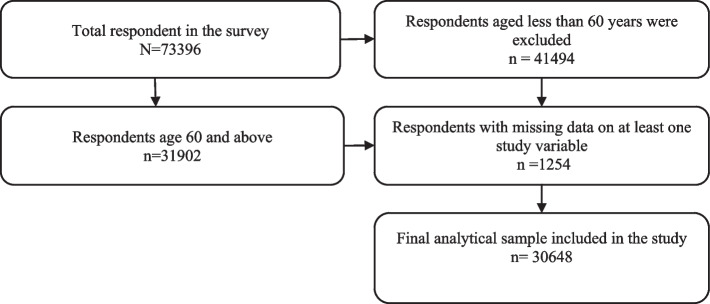
Selection criteria of the sample

The data for this study were obtained from the first wave of the Longitudinal Aging Study in India (LASI), conducted in 2017–2018. This nationally representative survey includes over 72,000 elderly individuals aged 45 and older, along with their spouses, regardless of age, from all states and union territories of India. The survey employed a multistage stratified area probability cluster sampling design to ensure the representation of the target population. In the first stage, primary sampling units (PSUs) were selected at the sub-district level within each state and union territory. The second stage involved selecting villages in rural areas and wards in urban areas within the chosen PSUs. In urban regions, an additional step was included: a census enumeration block (CEB) was randomly selected from each urban area, followed by the selection of households from each CEB in the fourth stage. This study specifically focuses on elderly adults aged 60 and above, comprising a sample of 30,648 respondents [23]. Fig. 1: show the flow chart of inclusion and exclusion criteria for sample selection in this study.

### Outcome variable

The LASI survey employs a five domain approach to assess cognitive functioning among older adults, with each domain comprising specific tasks and associated score ranges. Memory is evaluated using immediate word recall (0–10) and delayed word recall (0–10). Orientation includes questions on time (0–4) and place (0–4). Arithmetic function is assessed through backward counting (0–2), serial 7 subtractions (0–5), and simple computation tasks (0–2). Executive function is measured using paper folding (0–3) and pentagon drawing (0–1). Object naming is assessed (0–2).The composite cognitive score is calculated by summing the scores across all domains, resulting in a total score ranging from 0 to 43. Higher scores indicate better cognitive functioning, while lower scores suggest greater cognitive impairment. To categorize cognitive status, individuals falling below the 10th percentile of the composite cognitive score distribution were classified as having cognitive impairment coded as ‘1’ while those above this threshold were considered to have no cognitive impairment coded as ‘0’following the criteria suggested by [28].

### Predictor variables

To assess substance use, two primary variables were considered. Ever alcohol consumption was measured using the LASI survey question “Do you drink alcohol?” with binary response options “Yes” and “No”. Similarly, information on smoking behaviour was captured through the question “Have you ever smoked tobacco (cigarette, bidi, cigar, hookah, cheroot) or used smokeless tobacco (such as chewing tobacco, gutka, pan masala, etc.), also with response options ‘Yes’ and ‘No’. Age at first alcohol consumption was categorized as < 18 years, 18–45 years, and 45 + years. Activities of Daily Living (ADL) were assessed based on difficulties in dressing, walking across the room, bathing, eating, or using the toilet. A respondent was considered to have an ADL limitation if they reported difficulty in any of these tasks. Participation in yoga, pranayama, meditation, or asanas was categorized as “regular”, “sometime”, and hardly ever or never. Sleeping problems were recoded into a binary variable: those reporting 'never' were categorized as having no sleep problems, while all other responses indicated a sleep problem [42]. Based on WHO guidelines, respondents were classified as physically active if they engaged in at least 150 min of moderate-intensity activity, 75 min of vigorous intensity activity, or an equivalent combination per week. Those not meeting these thresholds were classified as physically inactive. This classification was derived from self-reported time spent on moderate and vigorous activities [5]. Food insecurity was assessed using four validated items from the FIES module in LASI, with responses indicating reduced meal size, hunger, fasting, or weight loss due to lack of food. Following established practice individuals reporting any affirmative response were categorized as food “insecure” while others were considered food “secure” [11].

Living arrangements were grouped into ‘living alone’, ‘living with spouse’, ‘living with spouse and children’ and ‘living with children only’. Body Mass Index (BMI) was categorized as ‘underweight’ ‘normal’ ‘overweight’ or obese. Self-rated health status was classified as good, fair, or poor. Age groups “60–69 years”, “70–79 years”, “80+ years” gender “male” or “female”, place of residence “urban” or “rural” and social group “Scheduled Caste”, “Scheduled Tribe”, “Other Backward Class”, and “Others”. Marital status was classified as “not in union”, “currently married”, or widowed. Regions were categorized into six zones “North”, “Central”, “East”, “West”, “Northwest” and South. Educational attainment “no formal education”, “up to primary”, “secondary and above”, “graduate and above. Current working status “currently working”, “currently not working”, “never worked”, “retired” and monthly per capita consumption expenditure (MPCE) quintiles “Poorest”, “Poorer”, “Middle”, “Richer” or Richest. By incorporating these diverse predictors, the study aimed to comprehensively examine the association between substance use and lifestyle with cognitive impairment, while accounting for a broad range of demographic and socioeconomic factors.

### Statistical analysis

In this study, we performed descriptive statistics to determine frequency distribution of both the explanatory variables and outcome variables. Subsequently, bivariate analysis (cross-tabulation) was performed with chi-square to understand the association between each predictor variable and the outcome variable. Additionally, binary logistic regression was employed to estimate the determinants of cognitive impairment among older adults. The results were presented in the form of an unadjusted odd ratio and an adjusted odd ratio with a 95% confidence interval. All the statistical analyses were performed with the help of STATA.

$$\text{ln}\left[\frac{\pi }{1-\pi }\right]={\beta }_{0}+{\beta }_{1}{X}_{1}+{\beta }_{2}{X}_{2}+{\beta }_{3}{X}_{3\dots \dots }{\beta }_{n}{X}_{n}+\varepsilon$$

where, ${\beta }_{0}$ is the intercept and ${\beta }_{1}$, ${\beta }_{2}$, and ${\beta }_{3}$, are the regression coefficients measuring the ${X}_{1}$, ${X}_{2} {X}_{3}$ relative effect of explanatory variables on mental cognition and ∈ represents the residual or unexplained variation in the model.

## Results

Table 1 presents data on the prevalence of cognitive impairment among older adults, categorized by substance use and lifestyle factors. The analysis reveals significant associations between Substance uses and cognitive impairment. In terms of substance use, the prevalence of cognitive impairment was higher among older adults who consumed alcohol (10%) compared to non-drinkers (9%). Moreover, those who initiated alcohol use before the age of 18 had a higher prevalence (13%) than those who started at age 45 or later (10%). Similarly, individuals who had ever smoked exhibited a higher prevalence of cognitive impairment (11%) than those who had never smoked (9%). Those who never used medication had the highest prevalence (11%), followed by occasional users (5%), while regular users had the lowest prevalence (4%). Individuals without sleep problems had a lower prevalence of cognitive impairment (9%) compared to those with reported problem (12%). Additionally, food insecurity was linked to cognitive decline, with higher prevalence among food insecure individuals (12%) compared to those who were food secure (8%). Regarding functional health, cognitive impairment was more common among those with limitations in Activities of Daily Living (ADL) (20%) than those without such limitations (8%). Individuals who self-rated their health as poor had a higher prevalence (15%) than those with good self-rated health (7%) Underweight older adults demonstrated greater cognitive impairment (14%) compared to those of normal weight (7%). Education was a strong determinant those with no formal education had (17%) compared with higher education (7%). Physical inactivity was also associated with a greater prevalence (13%) compared to those who were physically active (6%).

**Table 1:** Prevalence of cognitive impairment by substance uses, lifestyles, socio-economic and demographic characteristics of the older adult’s population, India, 2017–18

Variables	Cognitive Impairment	
	Sample(n)	Prevalence (%)
Alcohol consumption**		
No	5427	8.96
Yes	26,217	10.17
Age at first alcohol consumption		
Bellow 18 years	821	12.79
18–45	4112	7.39
45–84	230	10.01
Ever smoking **		
No	12,251	9.68
Yes	19,387	10.48
Medication***		
Never	26,855	11.09
Sometime/frequently	1324	4.53
Regular	3438	4.01
Sleeping Problem***		
No	17,687	8.87
Yes	14,109	12.17
Food security***		
Secure	16,913	8.56
Insecure	14,514	11.64
ADL***		
No	24,986	7.60
Yes	6787	20.11
Self-rated health***		
Good	16,809	7.07
Fair	10,067	9.06
Poor	4365	15.35
BMI***		
Underweight	6552	13.48
Normal Weight	14,907	7.60
Overweight	5296	3.81
Obese	1648	3.4
Physical activity***		
Inactive	22,518	12.53
Active	9384	6.08
working status		
currently working	9063	6.45
currently not working	11,076	13.45
never worked	9053	13.72
Retired	2710	3.99
Living arrangement***		
Living alone	1630	14.29
Living with spouse	8034	8.49
Living with spouse and children	13,697	6.04
Living with children	8541	16.64
Age Group**		
60–69	19,211	6.65
70–79	9250	12.38
80 +	3441	29.15
Gender***		
Male	15,340	6.82
Female	16,562	14.36
Residence***		
Rural	21,085	12.48
Urban	10,817	7.34
Caste **		
SC	5157	10.76
ST	5334	18.67
OBC	12,137	9.02
Others	9274	8.01
Marital status***		
Never married	845	14.79
Currently married	20,212	6.72
Widowed	1906	17.57
Region***		
North	5812	8.72
Central	4262	8.96
East	5757	11.15
Northeast	3752	13.41
West	4303	13.5
South	8016	10.1
Level of education***		
No formal education	17,191	16.56
up to primary	7648	5.22
secondary and above	5745	2.68
Graduation and above	1318	1.97
MCPE Quintile***		
Richest	5988	7.68
Richer	6259	8.53
Middle	6502	10.01
Poorer	6573	11.43
Poorest	6580	15.11
Total	30,648	5056(16.50)

The analysis includes an unequal sample size across the variables. Each category is represented by an unequal number of observations due to missing cases (30,648), Statistical significance levels indicating ***p < 0.001, **p < 0.01, and *p < 0.05

Sociodemographic disparities were also evident. Older adults who had never worked had a higher prevalence (14%) than those currently employed (6%). The prevalence was greater among individuals aged 80 and above (29%) than their younger counterparts (7%). Women exhibited a higher prevalence of cognitive impairment (14%) compared to men (7%), and rural residents had higher rates (12%) than urban dwellers (7%). Scheduled Caste respondents had significantly higher prevalence (19%) than other caste groups. Never married (15%) and widowed individuals (18%) had higher prevalence than those currently married (6%). Those living alone had a higher prevalence (15%) than those living with a spouse (8%). Economic status revealed disparities, with the poorest individuals showing higher prevalence (15%) than others. Underweight older adults demonstrated greater cognitive impairment (14%) compared to those of normal weight (7%). Education was a strong determinant those with no formal education had (17%) compared with higher education (7%).

### Determinants of cognitive impairments

Table 2 presents the results of binary logistic regression examining the determinants of cognitive impairment among older adults aged 60 years and above, using both unadjusted odds ratios (UOR) and adjusted odds ratios (AOR) with 95% confidence intervals.

**Table 2:** Logistic regression estimates for cognitive impairment by background characteristics among older adults in India, 2017–18

Variables	UOR (95%CI)	AOR (95%CI)
Alcohol consumption		
No ®		
Yes	1.17 (1.07,1.28) ***	1.07 (1.01,1.31) *
Age at first alcohol consumption		
Below 18 years ®		
18–45	0.54 (0.43,0.69) *	0.84 (0.53,1.34) *
46–84	0.76 (0.47,1.22) *	1.53 (0.63,3.67) *
Ever Smoking		
No ®		
Yes	1.04 (1.03,1.09) **	1.06 (0.76,1.15) **
Medication Use		
Regular®		
Sometime	1.08(0.79,1.47) *	0.96 (0.70,1,23) *
Never	2.85(2.40, 3.38) ***	2.10(1.93, 2,56) **
Sleeping Problem		
No®		
Yes	1.42(1.32,1.53) **	1.10(1.06, 1.20) **
Food security***		
Secure®		
Insecure	1.40(1.30,1.51) **	1.23(1.20, 1.37) **
ADL		
No ®		
Yes	3.41 (3.41,3.42) ***	1.54 (1.27,1.86) ***
Self-rated health		
good®		
Fair	1.37(1.25,1.50) ***	1.49 (1.25,1.78) ***
Poor	2.44(2.20,2.71) ***	2.36 (1.94,2.86) ***
BMI		
Normal weight®		
Underweight	3.90(3.33,4.57) ***	1.35 (1.14,1.61) ***
Overweight	2.05(1.76,2.39) ***	0.57 (0.43,0.75) **
Obese	0.79(0.59,1.09) *	0.91 (0.37,2.24) *
Physical activity		
Active®		
Inactive	2.07 (1.88,2.2) ***	1.67 (1.35,1.81) *
Working status		
Never work®		
Currently working	0.41 (0.37,0.45) ***	0.63 (0.48,0.82) ***
Currently not working	0.93 (0.86,1.01) ***	0.80 (0.66,0.98) ***
Retired	0.26 (0.21,0.32) **	0.71 (0.46,1.01) **
Living arrangement		
Living alone®		
living with spouse		0.78 (0.35,1.75) *
living with spouse and children		0.67 (0.3,1.49) *
living with children and other		1.17 (0.9,1.52) *
Age Group		
60–69®		
70–79		1.54 (1.29,1.84) ***
80 +		3.70 (3.03,4.53) ***
Gender		
Males®		
Females		1.95 (1.57,2.44) ***
Residence		
Urban®		
Rural		1.29 (1.03,1.62) ***
Caste		
Others®		
Schedule Caste		0.92 (0.75,1.14)
Schedule tribe		2.42 (1.87,3.13) ***
OBC		0.93 (0.76,1.13)
Marital status		
Never married®		
Currently married		0.76 (0.32,1.81)
Widowed		0.62 (0.4,0.96)
Region		
South®		
North		0.77 (0.60,0.99) *
Central		0.57 (0.43,0.74) ***
East		0.86 (0.69,1.08) *
Northeast		1.38 (1.04,1.82) ***
West		1.84 (1.38,2.46) ***
Education		
No formal education®		
up to primary		0.25 (0.18,0.33) ***
Secondary and above		0.18 (0.11,0.29) ***
Graduation and above		0.31 (0.1,0.93) ***
MCPE Quintile		
Poorest®		
Richest		0.71 (0.57,0.90) *
Richer		0.70 (0.52,0.94) *
Middle		0.64 (0.52,0.79) **
Poorer		0.79 (0.65,0.96) ***

Model 1 shows the *UORs* unadjusted odds ratios, whereas Model 2 provides the *AORs* adjusted odds ratios. *OR* Odds ratio, *ADL* activity of daily living, *BMI* body mass index ® = reference category, with statistical significance levels indicating ***p < 0.001, ** *p* < 0.01, and * *p* < 0.05

#### Model:1

The unadjusted analysis revealed that older adults who had ever consumed alcohol were significantly more likely to experience cognitive impairment than those who never consumed alcohol (OR:1.17, 95% CI: 1.08,1.78). Interestingly, those who initiated alcohol consumption before the age of 18 appeared to have lower odds of cognitive impairment than those who began drinking later, though this association was not statistically significant (OR: 0.76, 95% CI: 0.47,1.22). Older adults who had ever smoked had slightly higher odds of cognitive impairment compared to non-smokers (OR: 1.04, 95% CI: 1.03,1.09). Lifestyle practices also played a critical role. Individuals who never engaged in yoga, pranayama, or meditation had significantly higher odds of cognitive impairment compared to those who practiced regularly (OR: 2.85, 95% CI: 2.40,3.42). Sleep disturbances were significantly associated with cognitive impairment, with affected individuals showing 42% higher odds of impairment (OR: 1.42, 95% CI: 1.32,1.53). Similarly, food insecurity was a significant predictor, with affected individuals exhibiting 40% higher odds of cognitive decline (OR: 1.40, 95% CI: 1.30,1.51). Functional limitations in activities of daily living (ADL) were also associated with a substantially increased likelihood of cognitive impairment (UOR: 3.41, 95% CI: 3.41,3.42). Those who rated their health as poor had more than twice the odds of cognitive impairment compared to those with good self-rated health (OR: 2.44, 95% CI: 2.20,2.71). Underweight individuals showing nearly four times higher odds of cognitive impairment than those with normal weight (OR: 3.90, 95% CI: 3.33,4.57). Older adults not involved in physical activity had significantly higher odds of cognitive decline than those who were physically active (OR: 2.07, 95% CI: 1.88,2.00).

#### Model:2

After adjusting for potential confounders, the association between alcohol consumption and cognitive impairment remained significant, with older adults who consumed alcohol having more than three times the odds of impairment compared to non-drinkers (OR: 3.02, 95% CI: 1.75,5.21). Early initiation of alcohol use (before age 18) continued to show a higher, but not statistically significant, likelihood of impairment (OR: 1.53, 95% CI: 0.63,3.67). Smoking was weakly associated with cognitive impairment and was not statistically significant (OR: 1.06, 95% CI: 0.86,1.30). Individuals not engaging in yoga, pranayama, or meditation was significantly associated with increased odds of cognitive impairment (OR: 2.10, 95% CI: 1.93,2.56). Sleep disturbances remained a significant predictor (AOR: 1.10, 95% CI: 1.06,1.20), as did food insecurity (OR: 1.23, 95% CI: 1.20,1.37). Functional limitations in ADL continued to be strongly associated with cognitive decline (OR: 1.54, 95% CI: 1.27,1.86). Poor self-rated health was another key predictor, with such individuals having more than twice the odds of impairment (OR: 2.36, 95% CI: 1.96,2.86). Underweight status remained significantly associated with higher odds of cognitive impairment (OR: 1.35, 95% CI: 1.14,1.61). Lack of physical activity also remained a significant factor (OR: 1.67, 95% CI: 1.35,1.81), and never employed individuals continued to be at greater risk (OR: 0.63, 95% CI: 0.48–0.82).

In terms of demographic characteristics, older adults aged 80 years and above had substantially higher odds of cognitive impairment compared to younger age groups (OR: 3.68, 95% CI: 3.01,4.51). Female respondents had higher odds than their male counterparts (OR: 1.96, 95% CI: 1.03,1.62).Widowed or unmarried older adults had increased odds of cognitive impairment compared to currently married individuals (OR: 1.29, 95% CI: 0.55,3.08), although this result was not statistically significant. Rural residence was associated with 28% higher odds of cognitive impairment compared to urban areas (OR: 1.29, 95% CI: 1.03,1.62). Finally, educational attainment played a protective role, with individuals having no formal education showing substantially higher odds of impairment (OR: 0.25, 95% CI: 0.19,0.33).

## Discussion

This study highlights the significant role of substance use and lifestyle behaviours in shaping cognitive outcomes among older adults in India. Individuals reporting alcohol use had significantly higher adjusted odds of cognitive impairment compared to non-users, which conform to an earlier study by [17] [37]. Early initiation of alcohol consumption was especially detrimental, supporting evidence of its long term neurocognitive consequences. Similarly, ever smokers were more likely to exhibit cognitive decline, although this association was less pronounced after adjustment, possibly due to confounding by health status or comorbidities [12]. Conversely, protective lifestyle factors such as physical activity and medication use sleeping quality emerged as robust buffers against cognitive decline Even irregular adherence to medication was associated with lower odds of cognitive impairment, likely reflecting greater health awareness and access to healthcare [26]. Sleep disturbances also emerged as a major risk factor, aligning with prior evidence on the role of sleep in memory consolidation and brain health [42]. Food insecurity was another significant predictor, likely mediated by chronic stress and poor nutrition [35]. Functional limitations in activities of daily living (ADL) were independently associated with cognitive decline, suggesting a possible bidirectional relationship between cognitive and physical functioning [20]. Individuals engaging in regular physical activity demonstrated significantly lower odds of cognitive impairment [40]. Individuals who never socially engaged were more likely to experience cognitive impairment [6]. Additionally, reading habits were strongly associated with reduced odds of cognitive impairment [7]. Older adults who never participated in social activities were at increased risk, supporting the literature on social isolation and cognitive decline [44].

Social environment played a critical role. Older adults living with a spouse and children had significantly lower odds of cognitive impairment compared to those living alone, underscoring the benefits of intergenerational support [14]. Demographic patterns further revealed that the likelihood of cognitive impairment increases significantly with age, particularly among those aged 80 and above [47]. Gender differences were prominent, with older women showing nearly double the odds of cognitive impairment compared to men [1]. Rural residents experience greater cognitive impairment than their urban counterparts, potentially due to limited healthcare access, lower educational attainment, and reduced opportunities for cognitive stimulation [31]. Caste based and regional disparities in cognitive impairment were evident, with Scheduled Tribe populations exhibiting significantly higher risk. This may stem from long-standing marginalization, limited access to resources, and greater exposure to risk factors like alcohol use [25]. Marital status also influenced cognitive health widowed or divorced individuals had greater odds of cognitive impairment than married counterparts [16]. Higher educational attainment was significantly associated with lower odds of cognitive impairment, underscoring its protective role in late-life cognitive health [33]. Individuals in higher economic strata experienced lower odds of impairment, likely due to better access to healthcare, nutrition, and overall quality of life [4]. Poor self-rated health was also associated with greater cognitive impairment, reinforcing the importance of perceived health as a marker of broader wellbeing [24]. The major strength of this study lies in its use of a comprehensive cognitive score and a large, nationally representative sample of older adults, enabling robust multivariate analysis across a wide range of variables. This is the first study in India to examine substance use and cognitive impairment using a 0–43 composite cognitive score in conjunction with multiple lifestyle factors.

There are several limitations of this study. First, the cross sectional nature of the data limits our ability to infer causality between substance use, lifestyle factors, and cognitive impairment. Second, although cognitive impairment was assessed using a validated tool from LASI, the absence of clinical or neuropsychological diagnostic confirmation may affect the accuracy of classification, particularly given known limitations in sensitivity and specificity. Third, important covariates such as sedative or analgesic use, detailed physical health conditions, and chronic disease status were not included, which may introduce residual confounding. Fourth, while we included selected lifestyle variables such as medication use and sleep disturbances, other relevant dimensions like detailed dietary patterns or physical activity intensity were not incorporated due to data limitations. Fifth, substance use variables were dichotomized based on “ever use,” without accounting for type, frequency, duration, or intensity, which could obscure more granular associations. Finally, unmeasured factors such as environmental exposures, access to healthcare, or genetic predispositions could not be accounted for and may influence the observed associations.

## Conclusion

This study provides comprehensive evidence that substance use, particularly tobacco and alcohol consumption, is significantly associated with an increased risk of cognitive impairment among older adults in India. Moreover, several lifestyle and social determinants including physical inactivity, sleep disturbances, social disengagement, poor self-rated health, and food insecurity emerged as key risk factors for cognitive decline. Conversely, higher educational attainment, regular medication adherence, economic wellbeing, and engagement in cognitively stimulating activities such as reading and social interaction were found to have strong protective effects. These findings underscore the urgent need for context specific, multidimensional interventions targeting high risk populations especially older adults who are widowed, reside in rural areas, are economically disadvantaged, or engage in harmful substance use. Tailored cognitive screening and awareness efforts for these vulnerable groups may help mitigate long term impairment and improve overall well-being. The results also align with India’s commitment to achieving Sustainable Development Goal 3, which promotes healthy lives and wellbeing for all age groups.

## Data Availability

The study utilizes secondary data which is available only on request from [datacenter@iipsindia.ac.in](mailto:datacenter@iipsindia.ac.in).
